# Impact of COVID-19 Pandemic on Expenses of Medical Students Applying to Otolaryngology Residency Programs

**DOI:** 10.7759/cureus.28676

**Published:** 2022-09-01

**Authors:** Kristina F Powers, Nicole M Favre, Maya Raghavan, Michele M Carr

**Affiliations:** 1 Otolaryngology, Jacobs School of Medicine and Biomedical Sciences, University at Buffalo, Buffalo, USA

**Keywords:** u.s. medical residency, residency application process, covid 19, cost analyses, otolaryngology residency

## Abstract

Introduction: The coronavirus disease 2019 (COVID-19) pandemic has led to many changes in the residency application process. The purpose of this study was to determine the impact of these changes on the cost of applying to otolaryngology residency programs.

Materials and Methods: A retrospective, cross-sectional analysis was conducted using the Texas Seeking Transparency in Application to Residency (Texas STAR) Dashboard database to determine the differences in residency application costs from 2019 to 2022. Applicant information and cost data including application fees, interview expenses, away rotation expenses, total expenses, and geographic regions were collected. Median expenses and interquartile ranges were reported for each year and geographic region. Non-parametric comparisons were conducted.

Results: Data from 499 otolaryngology applicants were collected from the Texas STAR database. The total expenses, interview expenses, and away rotation expenses of applicants from 2019 to 2022 were significantly decreased (p < 0.001) in all regions of the United States with the greatest decrease between 2020 and 2021. Application fees (p = 0.005) were not significantly different among regions of the United States throughout the time period studied.

Conclusion: The COVID-19 pandemic significantly decreased the total expenses of applying to otolaryngology residencies including away rotation and interview expenses.

## Introduction

The coronavirus disease 2019 (COVID-19) pandemic has led to significant changes in the process of applying to residency programs in the United States, especially in the field of otolaryngology. At the onset of the COVID-19 pandemic in 2020, the Coalition for Physician Accountability discouraged students from attending subinternships at other institutions, better known as “away rotations” [1]. This was followed by a limit of one away rotation in 2021 due to the concerns about increasing COVID-19 prevalence, followed by unlimited away rotations in 2022 [1]. 

These restrictions led to a reduction in the financial burden of the residency application process as each outside rotation costs on average $1000, students were no longer able to evaluate potential residency programs or “audition” at a program to facilitate matching [2]. Away rotations are an important part of the residency application process, specifically in otolaryngology. The average otolaryngology residency applicant completes 1.8 away rotations and 36% reportedly match into one of the programs where they rotated [3]. In response to the concerns about limiting away rotations, virtual away rotations were implemented in addition to otolaryngology-specific programs such as Short Talks by Aspiring Residents (STAR) Oto and preference signaling [2, 4-5].

At the start of the pandemic, a signaling program was implemented to allow applicants to demonstrate interest in a program in lieu of away rotations [6]. Applicants were able to signal a limited number of programs before submitting their residency applications to increase their chances of receiving an interview [6]. Similarly, a program called STAR Oto was created to allow medical students to virtually share short bibliographical and academic talks with various otolaryngology programs around the country and obtain mentorship from participating faculty during the pandemic [5]. At the beginning of the pandemic, the Association of American Medical Colleges (AAMC) stated that all residency interviews should be transitioned to a virtual platform, which has continued into the 2022-2023 application cycle [7]. Although this change financially benefited applicants, a recent study indicated that otolaryngology applicants who were interviewed virtually were significantly more likely to match within their geographic region as compared to prior to the pandemic [8]. 

As a result of the numerous changes implemented throughout the COVID-19 pandemic, there was a significant transition to virtual platforms. To our knowledge, no studies have evaluated the financial savings associated with the implemented changes specifically in relation to otolaryngology residencies. The implications of these changes are important to evaluate despite decreased opportunities for applicants because the decreased cost of applications may have enabled lower income students to be more competitive in the pursuit of otolaryngology residency positions [9]. The purpose of this study is to compare the cost of the residency application process including interviews, applications, away rotations, and total costs before and during the COVID-19 pandemic.

## Materials and methods

The Texas Seeking Transparency in Application to Residency (Texas STAR) Dashboard database was used to conduct a retrospective, cross-sectional analysis of the differences in otolaryngology residency application costs between 2019, 2020, 2021, and 2022. Texas STAR is a nationwide survey and online tool used to help medical students and advisors to navigate the constantly evolving match process [10]. This database is the largest and most current nationwide survey dataset and is the only resource available that compiles all associated expenses involved in the transition from medical school to residency [10]. Fourth-year medical students from participating medical schools completed an annual survey about their own application experience including board examination scores, class quartiles, membership in honorary societies, publications, and volunteer experiences. Invitations to participate were sent immediately following the residency application cycle. The survey also included questions about the costs of different parts of the application process.

Our data pool consisted of applicants from 123 US allopathic medical schools who comprised 499 otolaryngology residency applicants. Osteopathic and international medical graduate statistics were not available for inclusion. The results were comparable to the National Residency Match Program’s objective characteristics, providing assurance of the validity of the dataset. The data from this tool is de-identified and publicly available; therefore, this study did not need institutional review board approval. 

Statistical analysis

We compared applicant characteristics between the four years, with 2019 and 2020 being prior to COVID-19 modifications, and 2021 and 2022 being after the changes. In addition, we compared each of the expense categories between the four years and compared costs based on geographic region. Median expenses and interquartile ranges are reported for each year and geographic region. For statistical analysis, non-parametric comparison using the Kruskal-Wallis H test was conducted to determine if there was a difference in median expenses based on year and geographic region.

## Results

Demographics of applicants are shown in Table 1. Cost data were obtained and included: application fees, interview expenses, away rotation expenses, and total expenses. The data were separated into five geographic regions.

**Table 1:** Demographics of otolaryngology residency applicants by year. CGSA, Central Group on Student Affairs; NEGSA, Northeastern Group on Student Affairs; SGSA, Southern Group on Student Affairs; WGSA, Western Group on Student Affairs; AOA, Alpha Omega Alpha Honor Society

	2019 (N=105)	2020 (N=143)	2021 (N=115)	2022 (N=136)
Number of applicants from CGSA N (%)	25 (24)	38 (27)	25 (22)	25 (18)
Number of applicants from NEGSA N (%)	27 (26)	33 (23)	34 (30)	42 (31)
Number of applicants from SGSA N (%)	44 (42)	58 (41)	49 (43)	44 (32)
Number of applicants from WGSA N (%)	9 (9)	14 (10)	7 (6)	22 (16)
Number of matched applicants N (%)	86 (82)	124 (87)	105 (91)	105 (77)
Mean step 1 score	247	248	247	248
Mean step 2 score	255	256	256	254
Percentage of applicants in AOA	44	37	42	43
Mean number of interview invites	18	16	13	12
Mean number of interviews attended	12	12	12	11
Mean number of publications	4	5	5	5
Mean number of research experiences	6	6	7	7
Mean number of presentations	7	6	8	8
Mean number of clerkships honored	5	4	4	4
Mean number of volunteer experiences	7	8	8	5
Mean number of leadership positions	4	5	5	6

Table 2 summarizes the median expenses based on geographic region and year. The total expenses, interview expenses, and away rotation expenses of applicants were significantly decreased (p < 0.001) in all regions of the United States during 2019 to 2022 with the greatest decrease between 2020 and 2021. This trend was not seen in application fees which fluctuated throughout the years included in this study. There was an overall increase in application fees in most regions of the United States in 2022.

**Table 2:** Comparison of application, interview, away rotation, and total expenses by geographic regions and years in USD. Data presented as median with 95% CI CI, confidence interval; USD, United States dollars *p value determined by Kruskal Wallis

Application fees in USD						
	2019 (N=105) Median (range)	2020 (N=143) Median (range)	2021 (N=115) Median (range)	2022 (N=136) Median (range)	All years (499) Median (range)	Difference in cost by year p value*
Central	1500 (1250-1750)	1750 (1750-1750)	1250 (1250-1750)	1750 (1750-2250)	1750 (1750-1750)	0.002
Northeast	1250 (1250-1250)	1750 (1250-1750)	1250 (1250-1750)	1750 (1750-1960)	1500 (1250-1960)	< 0.001
South	1750 (1250-1750)	1750 (1591-1750)	1750 (1750-1750)	1750 (1750-2250)	1750 (1750-1750)	0.07
West	1750 (1750-1750)	1250 (1250-1750)	1250 (1250-1750)	2250 (1750-2250)	1750 (1750-1750)	0.001
Difference in cost by region (p value)*	<0.001	0.05	0.4	0.01	0.005	
Interview expenses in USD						
Central	2750 (2250-3250)	2750 (2250-3750)	250 (250-250)	250 (250-250)	250 (250-1250)	< 0.001
Northeast	2250 (1750-2750)	2750 (2250-3250)	250 (250-250)	250 (250-250)	250 (250-750)	< 0.001
South	2750 (2750-3750)	3250 (2750-4750)	250 (250-250)	250 (250-250)	750 (250-1250)	< 0.001
West	3250 (3250-3750)	2750 (2250-3250)	250 (250-250)	250 (250-250)	250 (250-750)	< 0.001
Difference in cost by region (p value)*	< 0.001	0.08	1	0.5	0.2	
Away rotation expenses in USD						
Central	1750 (1250-1750)	1750 (1250-2250)	250 (250-750)	1750 (1750-2250)	1250 (1250-1750)	< 0.001
Northeast	1750 (1591-2409)	2250 (1750-2250)	250 (250-250)	1750 (1250-2176)	1250 (1250-1750)	< 0.001
South	1750 (1750-2250)	2250 (1750-2750)	250 (250-250)	1750 (1324-2250)	1250 (1250-1750)	< 0.001
West	4250 (2750-4250)	1750 (1250-2250)	750 (750-750)	1750 (1250-2409)	1750 (1250-1750)	< 0.001
Difference in cost by region (p value)*	< 0.001	0.009	< 0.001	0.9	< 0.001	
Total expenses in USD						
Central	6250 (5750-6750)	6250 (5250-7750)	2250 (1750-2750)	3250 (3250-4250)	4750 (4250-5250)	< 0.001
Northeast	5750 (4825-6250)	6750 (6250-7750)	1750 (1750-1750)	3250 (3250-3750)	1750 (1250-1750)	< 0.001
South	7250 (5913-8250)	7750 (5750-8510)	2250 (1750-2250)	3750 (3250-4750)	4250 (3750-4750)	< 0.001
West	9750 (7750-10250)	7250 (6250-7750)	2250 (1750-2250)	3750 (3250-4750)	4750 (4250-5750)	< 0.001
Difference in cost by region (p value)*	< 0.001	0.7	<0.001	0.08	0.004	

There was a significant difference in total expenses (p = 0.004) between regions in the United States with decreased total expenses in the Northeastern United States compared to the other regions. There was also a significant difference in away rotation expenses (p < 0.001) between all regions of the United States with increased cost in the West compared to all other areas. This difference was most pronounced in 2019. There was no difference in the cost of away rotations between regions of the United States in 2022. Overall, there was no significant difference in the cost of interview expenses between regions (p = 0.2). In 2019, the interview expenditures were greatest in the West and in 2020 they were greatest in the South. In 2021 and 2022, all regions in the United States had the same median cost. Application fees (p = 0.005) were not significantly different among regions of the United States throughout the time period studied.

## Discussion

With the increased competitiveness of otolaryngology residency programs along with multiple changes to the application process during the time of the COVID-19 pandemic, it is important to determine their impact on applications and residency programs. Previous studies of otolaryngology applicants have used the National Residency Match Program data to show a significant increase in the number of applicants matching at or close to home during the years of the COVID-19 pandemic, likely due to the limitations of away rotations and in-person interviews [11-12]. Studies were performed with dermatology and orthopedics applicants looking more specifically at the change in costs related to the COVID-19 pandemic [9, 13]. These studies both found that virtual interviews and a limited number of away rotations significantly reduced the cost of the application process, with applicants from the Western United States medical schools potentially saving the most [9, 13]. To our knowledge, this is the first comparative cost analysis study conducted to evaluate the effect of the pandemic on otolaryngology applicants.

During the entirety of the time period analyzed in our study, there was a significant difference in cost when comparing expenses from each year. A decrease in costs before and after 2020 aligns with the beginning of the COVID-19 pandemic, likely related to the absence of away rotation expenses. A reduction in the cost of interview expenses likely also contributed to the total reduction in costs. Interview-related costs were down from greater than $2,000 to a mean of $250 after the AAMC stated that all residency interviews should be transitioned to a virtual platform from 2020 to 2023 [7]. There is no apparent trend in application fees from 2019 to 2022. In all regions of the United States there are fluctuations in application fees not related to the timing of the pandemic. This is likely because the residency application process including the online platform and application fees was largely unchanged as a result of the pandemic [14]. There was no significant difference in number of applications submitted, interviews attended, or number of programs ranked as a result of the pandemic [14].

A 2016 survey found that of all those who applied to otolaryngology residency programs at Dartmouth-Hitchcock Medical Center and MedStar Georgetown University Hospital, 28% did not have sufficient funds for applying and interviewing despite seeking out additional monetary resources [15]. The applicant’s region of the United States had an impact on this cost burden. In 2019, there was a significant difference in both application fees and interview expenses among regions with the northeast having a significantly lower average cost and the south and west having a significantly higher average cost. Gordon et al. showed a similar pattern in application and interview fees in orthopedic surgery, with the applicants in the northeast spending less on interview expenses but more on application fees. This could be a result of the greater number of residency programs located in the northeast, reducing travel expenses for applicants living in this area [9]. Since this cost difference dissipated when the pandemic started in 2020, the pandemic may have had a role in equalizing application cycle costs for applicants. Particularly, due to virtual interviews, cost has become less of a barrier for applicants who obtain interviews outside of their home geographic region. Applicants residing in the west had a significantly higher cost burden for away rotations compared to those in the other three geographic regions in 2019. This difference did not exist in the 2021-2022 application cycle, likely due to the AAMC limiting each applicant to one away rotation [7].

Limitations

This study is limited by the nature of anonymous public data collection. Otolaryngology applicants self-reported their data online with no means of verifying the accuracy. Additionally, not all applicants chose to share their information with Texas STAR which may have led to a biased sampling of applicants. It is possible that stronger applicants who successfully matched into a residency program chose to report their data, especially given that the Texas STAR survey was released to students after the conclusion of The Match. The data analysis is also limited by the information provided by the database. Cost was only reported via mean values with limited categories of expenses. It is possible that the categories did not accurately reflect all expenses that the applicants encountered during the application cycle, however, the most common expenditures that fourth year medical students faced were included in the Texas STAR survey.

## Conclusions

The COVID-19 pandemic had a significant impact on many aspects of the application process in the field of otolaryngology. Here, we see that the pandemic significantly decreased the cost of application fees, away rotation expenses and interview expenses, likely due to the move toward virtual interviews and a limited number of away rotations. While this is financially beneficial, further analysis needs to be done to determine how these decreased expenses and changing processes affect candidates’ chances of matching, satisfaction with their match results, and programs’ ability to attract the best candidates.

## References

[1] (2022). The coalition for physician accountability's work group on away rotations - faqs. https://www.aamc.org/what-we-do/mission-areas/medical-education/away-rotations-faqs.

[2] Mikhail D, Margolin EJ, Sfakianos J (2021). Changing the status quo: developing a virtual sub-internship in the era of COVID-19. J Surg Educ.

[3] Winterton M, Ahn J, Bernstein J (2016). The prevalence and cost of medical student visiting rotations. BMC Med Educ.

[4] Pletcher SD, Chang CW, Thorne MC, Malekzadeh S (2022). The otolaryngology residency program preference signaling experience. Acad Med.

[5] (2022). Star oto - short talks by aspiring residents in otolaryngology. https://www.vumc.org/ent/staroto.

[6] (2022). Otolaryngology preference signaling - otolaryngology program directors. https://opdo-hns.org/mpage/signaling.

[7] (2022). AAMC interview guidance for the 2022-2023 residency cycle. AAMC.

[8] Whisonant CT, Shahriari SR, McDonald CD, Moya AN, Ederle A, Borah G (2022). COVID-19 and the otolaryngology match: an increase in applicants remaining close to home. Cureus.

[9] Gordon AM, Conway CA, Sheth BK, Magruder ML, Vakharia RM, Levine WN, Razi AE (2022). How did coronavirus-19 impact the expenses for medical students applying to an orthopaedic surgery residency in 2020 to 2021?. Clin Orthop Relat Res.

[10] (2022). Texas star: medical school student affairs. https://www.utsouthwestern.edu/education/medical-school/about-the-school/student-affairs/texas-star.html.

[11] Bernstein JD, Harmon M, Watson D (2022). COVID-19 and the otolaryngology residency match: rising incidence of home matches. Laryngoscope.

[12] Mulcahy CF, Terhaar SJ, Boulos S, Lee E, Zapanta PE (2022). Did more otolaryngology residency applicants match at their home institutions in 2021? Investigating the impact of the COVID-19 pandemic. Ann Otol Rhinol Laryngol.

[13] Gorgy M, Shah S, Arbuiso S, Cline A, Russo M (2022). Comparison of cost changes due to the COVID-19 pandemic for Dermatology residency applications in the USA. Clin Exp Dermatol.

[14] (2022). Fees for eras® residency applications. Students & residents. http://residents.aamc.org/applying-residencies-eras/fees-eras-residency-applications.

[15] Polacco MA, Lally J, Walls A, Harrold LR, Malekzadeh S, Chen EY (2017). Digging into debt: the financial burden associated with the otolaryngology match. Otolaryngol Head Neck Surg.

